# Retinoic acid modulates peritoneal macrophage function and distribution to enhance antibacterial defense during inflammation

**DOI:** 10.3389/fnut.2025.1545720

**Published:** 2025-04-30

**Authors:** Yujuan Qin, Xi Wang, Xiamin Zhang, Lianting Nong, Qiyan Hou, Yuhong Chen, Yuting Li, Wenxian Lin, Xiuli Mao, Kezhao Wu, Wenqian Nong, Tonghua Wang, Lingzhang Meng, Jian Song

**Affiliations:** 1 Graduate School, Youjiang Medical University for Nationalities, Baise, China; 2 Institute of Cardiovascular Sciences, The People’s Hospital of Guangxi Zhuang Autonomous Region & Guangxi Academy of Medical Sciences, Nanning, China; 3 School of Medicine, Guangxi University, Nanning, China; 4 Department of Digestive Diseases, Affiliated Hospital of Youjiang Medical University for Nationalities, Baise, China; 5 Institute of Physiological Chemistry and Pathobiochemistry, University of Münster, Münster, Germany

**Keywords:** peritonitis, large peritoneal macrophages, small peritoneal macrophages, retinoic acid, nanoparticles

## Abstract

**Background:**

Peritoneal macrophages, comprising large peritoneal macrophages (LPMs) and small peritoneal macrophages (SPMs), play a vital role in maintaining immune defenses during inflammation. However, the molecular mechanisms governing their responses, particularly the impact of retinoic acid (RA), remain poorly understood. This study aims to elucidate the role of RA in modulating macrophage function, distribution, and immune responses during bacterial infections.

**Methods:**

A murine model of peritonitis was established using *Escherichia coli* expressing a tdTomato fluorescence marker. The effects of RA on macrophage phagocytic capacity, population dynamics, and transcriptomic profiles were assessed using immunofluorescence, flow cytometry, RNA sequencing, and quantitative PCR. Additionally, RA-loaded ZIF-8 nanoparticles were employed to investigate the sustained effects of RA delivery.

**Results:**

RA significantly enhanced macrophage phagocytic activity, delayed functional decline, and promoted the recruitment of SPMs in the peritoneal cavity. Transcriptomic analysis revealed upregulation of leukocyte migration and cell adhesion pathways in RA-treated SPMs. RA treatment also induced distinct gene expression profiles in macrophage subpopulations, reflecting its role in immune modulation. Notably, RA-loaded ZIF-8 nanoparticles prolonged RA retention within macrophages, sustaining its effects.

**Conclusion:**

RA enhances antibacterial defense by modulating macrophage activity, providing new insights into immune regulation. These findings underscore the therapeutic potential of RA and its nanoparticle formulations in managing bacterial infections and inflammation.

## Introduction

Two distinct subpopulations of macrophages have been identified within the peritoneal cavity. The first is a subpopulation of large peritoneal macrophages (LPMs), which have a large morphology and constitute approximately 90% of peritoneal cavity macrophages (1, 2). The majority of macrophages are LPMs, which express high levels of F4/80 but low levels of MHC-II. This classification is based on the expression of cell markers, specifically CD11b, F4/80, MHCII, and GATA6. The remaining subpopulation is that of the smaller macrophages, which occupy approximately 10% of the peritoneal cavity. The remaining subpopulation comprises a smaller macrophage within the peritoneal cavity, representing approximately 10% of the LPMs. This is the small peritoneal macrophage (SPMs). SPMs express low levels of F4/80, but high levels of MHC-II, and their surface markers are CD11b^+^/F4/80^LOW^/MHCII^+^/GATA6^−^ (1). The LPM is currently the more extensively researched of the two, whereas the SPMs remains relatively understudied (1, 3–5).

The transcription factor GATA6 is indispensable for the sustenance of the LPMs community. It preserves the cellular state in a non-autonomous manner and is induced by retinoic acid (RA), which is produced by the metabolism of vitamin A within the peritoneal cavity (6, 7). RA production is dependent on the metabolic enzymes RALDH1 and RALDH2, the activity of which is essential for RA synthesis. The fact that these enzymes are produced by large omental mesothelial cells and mesenchymal fibroblasts in the peritoneal cavity, which express the transcription factor Wilms’ tumor 1 (WT1) (8), and retinoic acid induces and reversibly regulates gene expression of GATA6 and other PMSGs in peritoneal macrophages. Chronic deficiency of vitamin A (retinoic acid precursor) leads to decreased expression of GATA6 in LPMs, triggering an inflammatory response and leading to the disappearance of LPMs. RA selectively affects the function and distribution of LPMs by regulating the expression of the transcription factor GATA6, a signature transcription factor of LPMs involved in the regulation of their gene expression profiles and in the maintenance of an anti-inflammatory phenotype (9). In the presence of RA, GATA6 upregulation was able to enhance the resistance of LPMs to infection and promote cell survival. This fact highlights the central role of the peritoneal microenvironment in RA generation and LPMs population maintenance. On the other hand, it is believed that SPMs are generated as macrophages by recruiting monocytes, which are the predominant cells in the peritoneal cavity as they respond to inflammation (5, 10, 11). Nevertheless, the rationale behind the presence of SPMs in the absence of inflammation and the potential role of retinoic acid in the peritoneal microenvironment in SPM recruitment under steady-state conditions remain uncertain Louwe et al. (12).

In the event of peritoneal cavity inflammation, both macrophage types undergo significant alterations, with the LPM disappearing rapidly in response to the inflammatory stimulus (13). This phenomenon is known as the macrophage disappearance reaction (MDR). The MDR is initiated when the LPMs undertakes bacterial containment by aggregating on the surface of the cavity or by aggregating in fibrous clots (14). A considerable number of LPMs undergo pyroptosis following aggregation and die, thereby rendering local proliferation an inadequate means of recovering the same number of LPMs that existed prior to the aggregation (15). In contrast, SPMs proliferate and become the predominant population in the peritoneal cavity during the inflammatory process (11, 12, 16). It is therefore generally accepted that SPMs are recruited and transformed from blood mononuclear cells. However, the transcriptomic, functional and phenotypic profiles of LPMs and SPMs during peritonitis remain unclear. Furthermore, the series of infectious events caused by peritonitis ultimately results in increased omental fibrosis, which in turn leads to the destruction of WT1^+^ mesothelium and mesenchymal fibroblasts, thereby reforming the peritoneal microenvironment conducive to RA production and the LPMs/SPMs compartment. Therefore, it is of clinical relevance to understand the role of RA for LPMs and SPMs in the inflammatory state.

The aim of this study was to investigate the response of peritoneal macrophages to bacterial attack in the presence of RA and changes in their immune defenses. Retinoic acid, a metabolite of vitamin A, has been shown to play a key role in the regulation of several immune functions. However, its specific effects on macrophage population dynamics and function under conditions of abdominal infection are unclear. To this end, we used pUC19-tdtomato to establish a peritonitis model with transduced bacteria and investigated the effects of RA on phagocytosis and on LPMs and SPMs macrophage populations by quantitative analysis and transcriptome sequencing. Our results suggest that RA may alter the distribution and function of macrophages in the peritoneal cavity by regulating their migration-related genes. At the early stage of infection, RA not only enhanced the phagocytic capacity of macrophages, but also significantly delayed the decline of their phagocytic function. In addition, RA-treated macrophages showed up-regulation of key genes GATA6 and RA-responsive genes, which was accompanied by significant changes in the cell migration-related transcriptome. In particular, changes in key genes related to migration function may play an important role in the dynamics of LPMs and SPMs populations. These findings reveal potential mechanisms of RA in regulating the antimicrobial function of abdominal macrophages. These findings provide important insights into the potential clinical applications of RA in infection control and other inflammatory disease therapies, and future studies may further explore the synergistic effects of RA with conventional anti-infective therapies.

## Materials and methods

### Mice

All C57BL/6J mice were obtained from the Guangxi Medical University Laboratory Animal Centre, and the animals were housed in the laboratory animal center under specific pathogen-free conditions, with feed and sterile water provided for the animals. The animals were kept using a 12 h light/12 h dark cycle.

### Peritonitis model and *E. coli*, RA injection

The experimental design included three different treatment groups to study the dynamic effects of RA and bacterial challenge on peritoneal macrophages:

#### Bacteria-only group

Mice were injected intraperitoneally with an *Escherichia coli* ER2272 strain harboring the pUC19-tdTomato construct (cultured overnight in LB at 37°C and then resuspended in 0.2 mL PBS to yield 1 × 10^7^ CFU). Samples were collected at 30 min, 60 min, and 4 h post-injection to assess baseline kinetics of bacterial uptake and macrophage response.

#### Only the RA group was injected

Mice were injected intraperitoneally with 200 μL of RA solution (10 μmol/mL). In this group, samples were also collected at 30 min, 60 min, and 4 h post-injection to assess the immediate distribution of RA in the peritoneal cavity and cellular uptake.

#### RA pretreatment with bacteria group

In another set of experiments, mice were injected once intraperitoneally with RA (200 μL, 10 μmol/mL) 24 h before bacterial challenge. 24 h later, these mice were injected with 1 × 10^7^ CFU *E. coli* Tdtomato in 0.2 mL of PBS. Subsequent samples were taken at the indicated time points for transcriptome sequencing.

### Immunofluorescence

The peritoneal mesothelium and greater omentum were excised and immediately embedded in Tissue-Tek O.C.T. compounds (Triangle Biomedical Sciences) and frozen in liquid nitrogen. They were then processed into 15-μm-thick sections. Following fixation in methanol at −20°C for 5 min, the frozen sections were rinsed on three occasions with PBS. Following a 40 min sealing process with PBS/1% BSA at room temperature, the sections were incubated with the antibody at a dilution of 1:1,000 at 4°C overnight. The images were captured using a ZEISS Axiovert 5 microscope.

### Flow cytometry

The peritoneal cavity was repeatedly flushed with 5 mL of ice-cold RPMI 1640 basal medium and cells were collected from the peritoneal exudate. Erythrocytes were lysed for antibody staining. All antibodies were incubated with the cells on ice at a 1:200 dilution for 15 min, then washed, resuspended, and analysis on a FACSC anto II (BD Biosciences) using FlowJo software.

### Real-time PCR

Peritoneal macrophages were extracted from the peritoneal cavity of mice. After 24 h of adhesion of macrophages to petri dish in complete medium containing 30% FBS, the macrophages were purified by washing away the non-adhesive cells and cultivated in serum-free basal medium supplemented with different drugs. For real-time PCR analysis, RNA was extracted using the TaKaRa RNAiso Plus kit. cDNA was reverse transcribed using Novozymes HiScript IV RT SuperMix for qPCR. Real-time PCR was performed on a QuantStudio5 detection system using SYBR green qPCR Master Mix (Table 1).

**Table 1:** Primers sequence.

Gene	Forward primer	Reverse primer
Apoc2	ctcggttcttcctggctctat	catgctgatcgggtatgtctt
Arg1	atggaagagtcagtgtggtgctg	tcaggagaaaggacacaggttgc
Cd62p	gaacctttgggtacaacagca	ttactgggaaccggaaactct
Cd73	agaaagttcgaggtgtggacat	cttcaggtagcccaggtatttg
Fn1	caagccacagtttctgatattcc	tctgctcctggtttaatgttgtt
Gata6	accatcaccatcacccgacctac	ctctccgacaggtcctccaacag
Icam2	acagctctgaaaaaggacggtct	gcagtattgacaccaccacgatg
Lrg1	agctatggtctcttggcagcatc	aattccaccgacagatggacagt
Rarb	acatgatctacacttgccatcg	tgaaggctccttctttttcttg
Serpinb2	gtgctgaagaagctagggaaaa	gttcacacggaaaggataaagc
Tgfb2	gtctcaacaatggagaaaaatgc	ctggttttcacaaccttgctatc
Thbs1	ggagatggaatcctcaatgaac	aagtgtcccctatgaggtctga
Cd49f	ctgaattcaaatgaagccaaaac	gactaattctgggatgccttttt

### Sorting peritoneal macrophages for RNAseq

To compared the effect of 24 h RA treatment on the SPMs and LPMs, we separated the mice into two groups: control and RA treatment. Upon the injection of bacteria containing pUC19-tdTomato, both SPMs and LPMs in the peritoneal cavity can be detected based on the antibody staining and phagocytosis of *E. coli*. SPMs and LPMs from the respective group were isolated from the peritoneal cavity using flow cytometry sorting. Total RNA was extracted from cell pellets using Trizol reagent according to the manufacturer’s protocol. The library was constructed using SMART-Seq_V4 Ultra Low Input RNA Kit for Sequencing. High-throughput RNA sequencing was performed using the Illumina NovaSeq Xplus platform, the raw data of which have been uploaded to GEO database.

### Bioinformatics analysis

Reference genome and gene model annotation files were directly downloaded from http://ftp.ensembl.org/pub/release-77/gtf/mus_musculus/. The index of the reference genome was built using Bowtie v2.2.3 and paired-end clean reads were aligned to the reference genome using STAR software. The alignment was converted to gene expression raw counts using feature counts and listed as a gene expression matrix, which can be included in the Supplementary data (on request). Prior to differential gene expression analysis, for each sequenced library, the read counts were adjusted by the edge R program package through one scaling normalized factor. Differential expression analysis of two conditions was performed using the DEG Seq R package (1.20.0). GSEA scoring of leukocyte migration was based on the hallmarker gene signature from GO database.

### ZIF-8 nanoparticles loaded with RA

Anhydrous ethanol was employed to solubilize 1 mg/mL ZIF-8 nanoparticles and RA individually, and the two were ultimately combined and loaded into the ZIF-8 Nanoparticles at 4°C. The loading amount of nanoparticles was tested for fluorescence intensity using a Thermo Fischer Multiscan Go Reader microplate reader under Alexa Fluor 488.

### Statistical analyses

One-way ANOVA and student *t test* were employed for the statistical analyses respectively, with a significance level of *p* < 0.05. The results of all replicate experiments are presented, with values expressed as Mean ± SEM.

## Results

**Figure 1:**
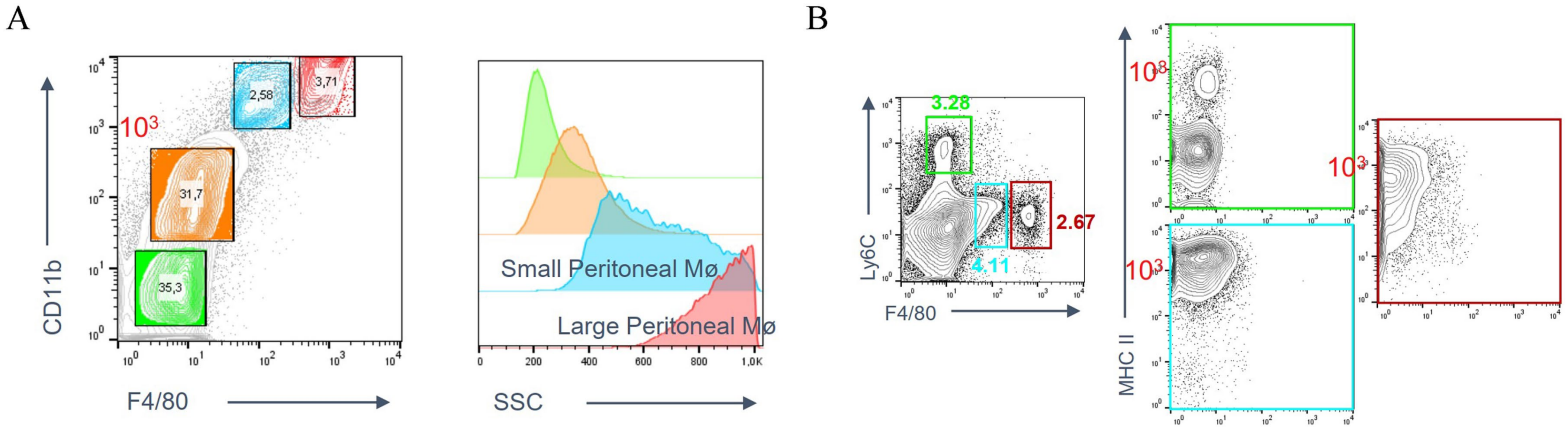
Peritoneal macrophage subpopulations. **(A)** Definition of LPMs and SPMs populations based on the expression of CD11b and F4/80 using flow cytometry. **(B)** Expression of by Ly6c and MHC II on LPMs and SPMs.

In flow cytometry analysis of peritoneal lavage fluid, different peritoneal cell populations were differentiated not only on the basis of their size (SSC), but also on the basis of their expression levels of CD11b and F4/80. These included: peripheral blood-derived monocytes, LPMs, and SPMs (Figure 1A). Large peritoneal macrophages and small peritoneal macrophages were subsequently subdivided on the basis of their expression of Ly6c and MHC II. LPMs were characterized by high expression of F4/80 and low expression of Ly6c, whereas SPMs were characterized by high expression of Ly6c and MHC II (Figure 1B).

**Figure 2:**
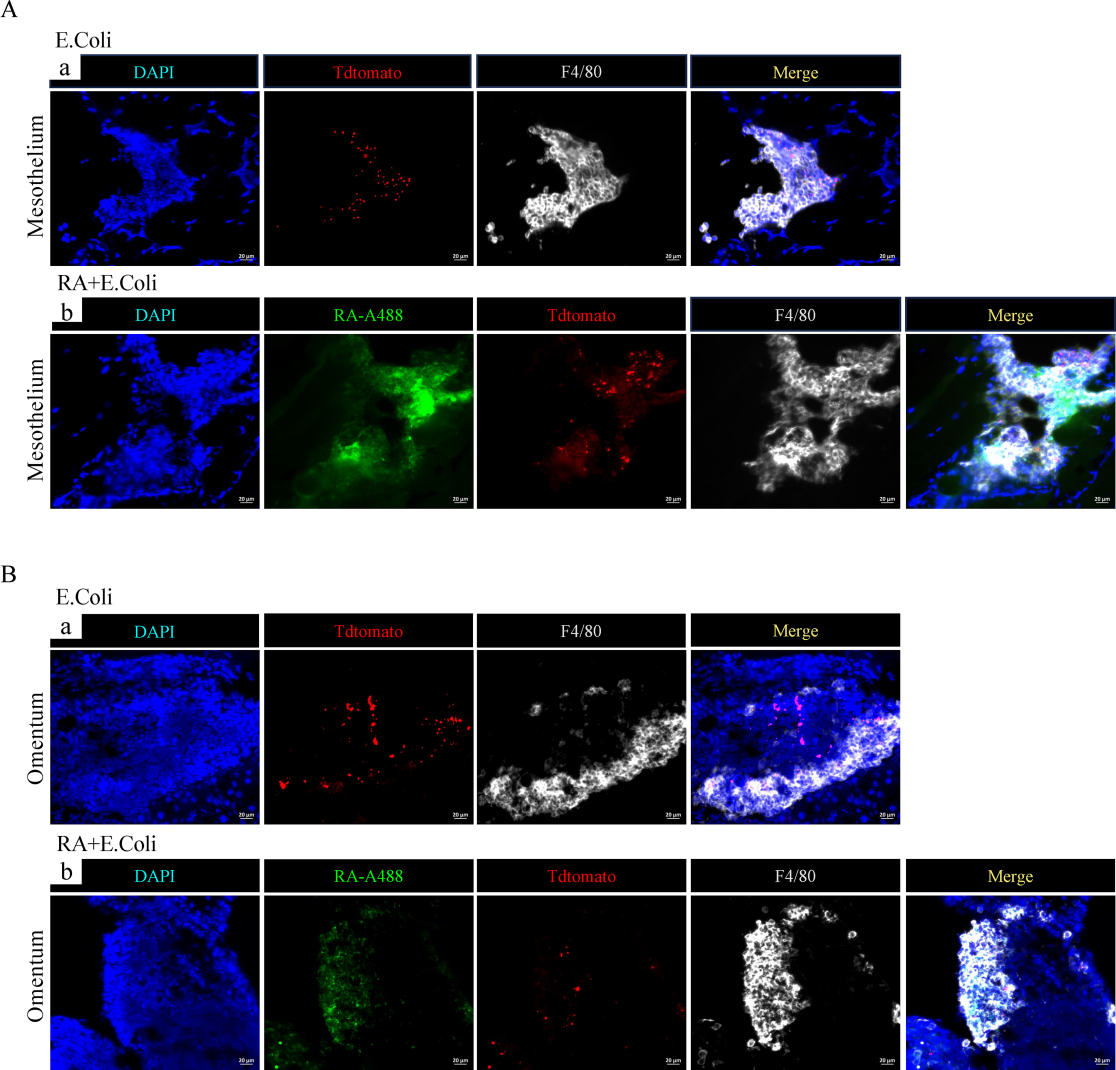
Immunofluorescence analysis of phagocytosis of RA and *E. coli* as well as the aggregates of macrophages on the peritoneal mesothelium and the greater omentum. **(A)** Immunofluorescence images of peritoneal mesothelial tissue showing the distribution of F4/80 staining (white), RA (green) and *E. coli* Tdtomato (red). **(B)** Immunofluorescence images of the greater omentum with F4/80 staining, RA and *E. coli* Tdtomato.

To study the impact of RA on the peritoneal macrophages during inflammation, a peritonitis model was established by using the *E. coli* Tdtomato strain. C57BL/6 mice were injected intraperitoneally with 1 × 10^7^ CFU of the *E. coli* Tdtomato strain. The peritoneal mesothelium and the greater omentum were observed to detect the aggregation of bacterial phagocytosis at 60 min post-injection. Also, we photographed the peritoneal mesothelium and the greater omentum after injections of Alexa Fluor 488 labeled RA. The results showed that RA was distributed around the macrophage aggregates in the mesothelium (Figure 2A) and the greater omentum (Figure 2B). After further injection of *E. coli*, clustering of RA, *E. coli* Tdtomato and F4/80 staining was observed in the aggregates, suggesting that RA may influence the local immune response by regulating macrophage migration and aggregation. Notably, cells phagocytosing high concentrations of RA did not completely overlap with cells phagocytosing large amounts of *E. coli*.

**Figure 3:**
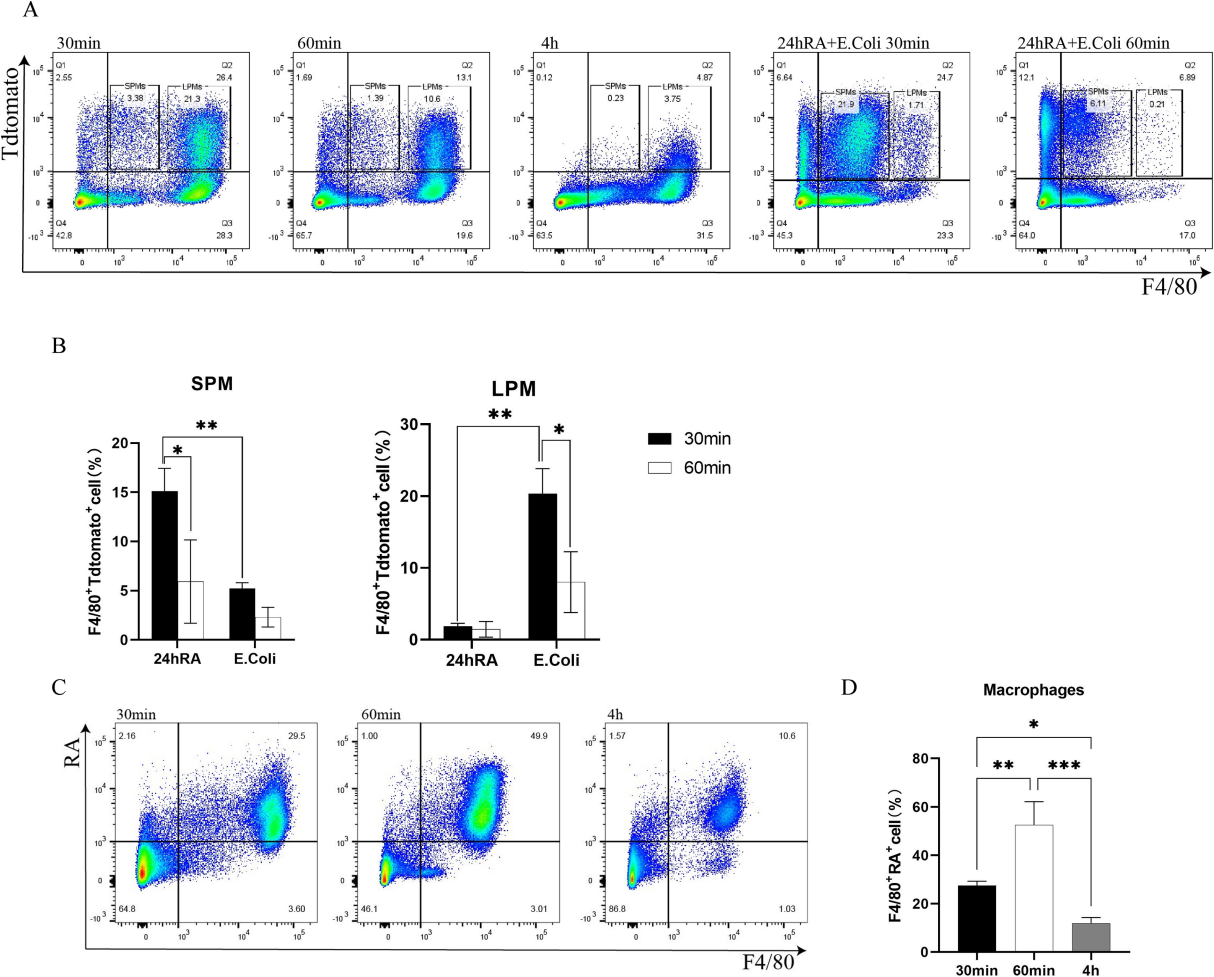
Flow cytometric analysis of macrophage phagocytosis of bacteria, RA uptake, and the effect of RA on phagocytosis of bacteria. **(A)** Phagocytosis of SPMs by macrophages in the peritoneal cavity of *Escherichia coli* and after 24 h of RA injection (24 h of RA pretreated group was not sampled after 4 h). **(B)** The ability of macrophages to phagocytose SPMs and LPMs bacteria was calculated separately 24 h after RA injection. Bars are expressed as mean ± SEM (*n* = 6) and significant differences were tested by t-test. **(C)** Phagocytosis of RA by peritoneal macrophages. **(D)** Quantitative determination of bacterial phagocytosis by macrophages after RA injection. Bar graphs are expressed as mean ± SEM (*n* = 6) and significant differences were calculated using one-way ANOVA.

To gain further insight into the impact of retinoic acid on peritoneal macrophages in the context of *E. coli* infection, a flow cytometry analysis was conducted based on two treatment groups: one group injected with only *E. coli*, and the other group injected with RA. The majority of F4/80+ macrophages were observed to phagocytose *E. coli* within 30 min. By 60 min, the number of F4/80+ macrophages that had phagocytosed bacteria had decreased substantially, with the majority of these cells disappearing by fourth (Figure 3A). However, the RA group was injected and demonstrated the ability to persist at the fourth hour (Figure 3C), indicating that macrophages phagocytosing RA are more stable in mice compared to macrophages phagocytosing bacteria. This indicates that RA is not rapidly metabolized or cleared. In particular, by the fourth hour, there was a notable decrease in cellular phagocytosis, which may have been attributed to a macrophage disappearance response to the substantial early depletion of macrophages. It was evident that retinoic acid reached its peak effect on macrophage activity at 60 min, after which a decline was observed (Figure 3D). This indicates that retinoic acid exerts a considerable influence on macrophage phagocytosis during the initial stages, although this impact may diminish over time. Additionally, we investigated the impact of RA on bacterial uptake by macrophages. The bacteria were injected 24 h after the administration of RA, and an increase in SPMs of F4/80 cells and a decrease in LPMs of F4/80 cells were observed (Figure 3B). This indicates that the recruitment or activation of macrophages may have been enhanced following RA treatment, resulting in an increased number of macrophages or a greater proportion of specific subpopulations within 24 h (Figure 3A).

**Figure 4:**
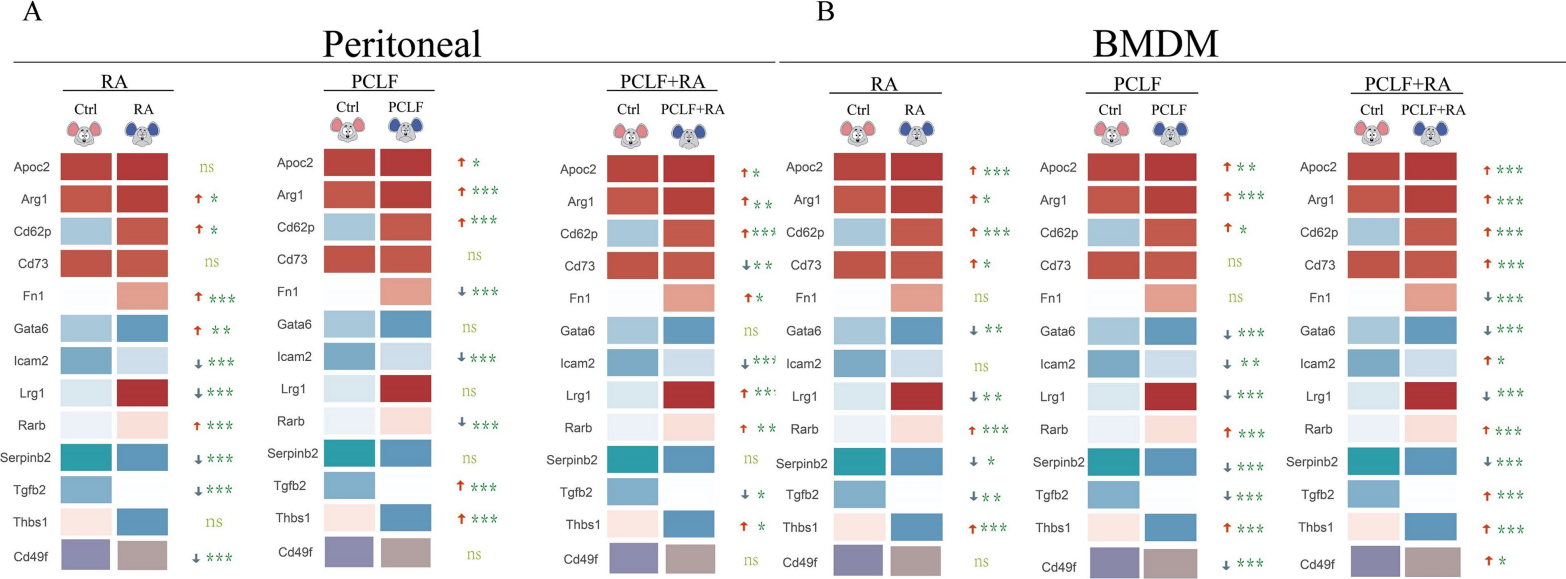
Distinct profiles of RA-response genes in peritoneal macrophages and BMDM. **(A)** In peritoneal macrophages, RA treatment significantly up-regulated the expression of Fn1, Gata6, Arg1, and Rarb (*p < 0.05). **(B)** In BMDM, RA treatment resulted in the up-regulation of the expression of the indicated genes. However, the trend observed for Rarb and Thbs1 differed from that seen in peritoneal macrophages. All data were subjected to one-way ANOVA for significance analysis, with *n* = 6 for each repeated experiment.

To elucidate the mechanism of retinoic acid action in macrophages of different origin, we performed quantitative polymerase chain reaction (qPCR) analysis of key gene expression in peritoneal macrophages (Figure 4A) and bone marrow-derived macrophages (BMDM, Figure 4B) under different treatment conditions. The aim of this experiment was to investigate the regulatory effects of RA on macrophage gene expression and the potential synergistic effects of intraperitoneal lavage fluid as an additional signaling source. The peritoneal macrophages were initially treated with RA and the peritoneal lavage, which served as an additional source of signals. The peritoneal lavage was prepared in a serum-free medium devoid of any vitamin A metabolite. This resulted in a notable induction of Arg1, Fn1, Gata6, and Rarb by RA, while the expression of CD49f, Icam2, Tgfb2, Lrg1, and serpinb2 was all found to be down-regulated. The observed alterations in gene expression suggest that RA may regulate macrophage activity and function by influencing cell adhesion, fibronectin production, intercellular signaling, and other biological pathways. In the peritoneal lavage-treated group, a trend of up-regulation was observed for Apoc2, Thbs1, Arg1, Cd62p, Tgfb2, and Thbs1. In the peritoneal lavage plus RA-treated group, Apoc2, Cd62p, Arg1, Lrg1, Rarb, and Thbs1 were found to be up-regulated, indicating that the components in the peritoneal lavage may have a coordinating effect, thereby further enhancing the expression of these genes (Figure 4A).

The same cultivation condition were employed to treat bone marrow-derived macrophages, and it was determined that Apoc2, Arg1, Cd62p, Cd73, Rarb, and Thbs1 were induced by RA. In the peritoneal lavage -treated group, an increase in the expression of Apoc2, Arg1, Cd62p, Cd73, Cd49f, Tgfb2, Thbs1, and Rarb was observed, indicating that the components in the peritoneal lavage may have a promotional effect on the expression of these genes in the BMDM (Figure 4B). The different effects of RA on peritoneal macrophages and BMDM may be related to their different origins and differentiation characteristics. LPMs, as peritoneal resident cells, may be more responsive to changes in local signaling molecules, whereas BMDM, due to their origin from myeloid cells, may be sensitive to systemic metabolic signals. These results further highlight the potential role of RA in regulating the function of different macrophage subpopulations.

**Figure 5:**
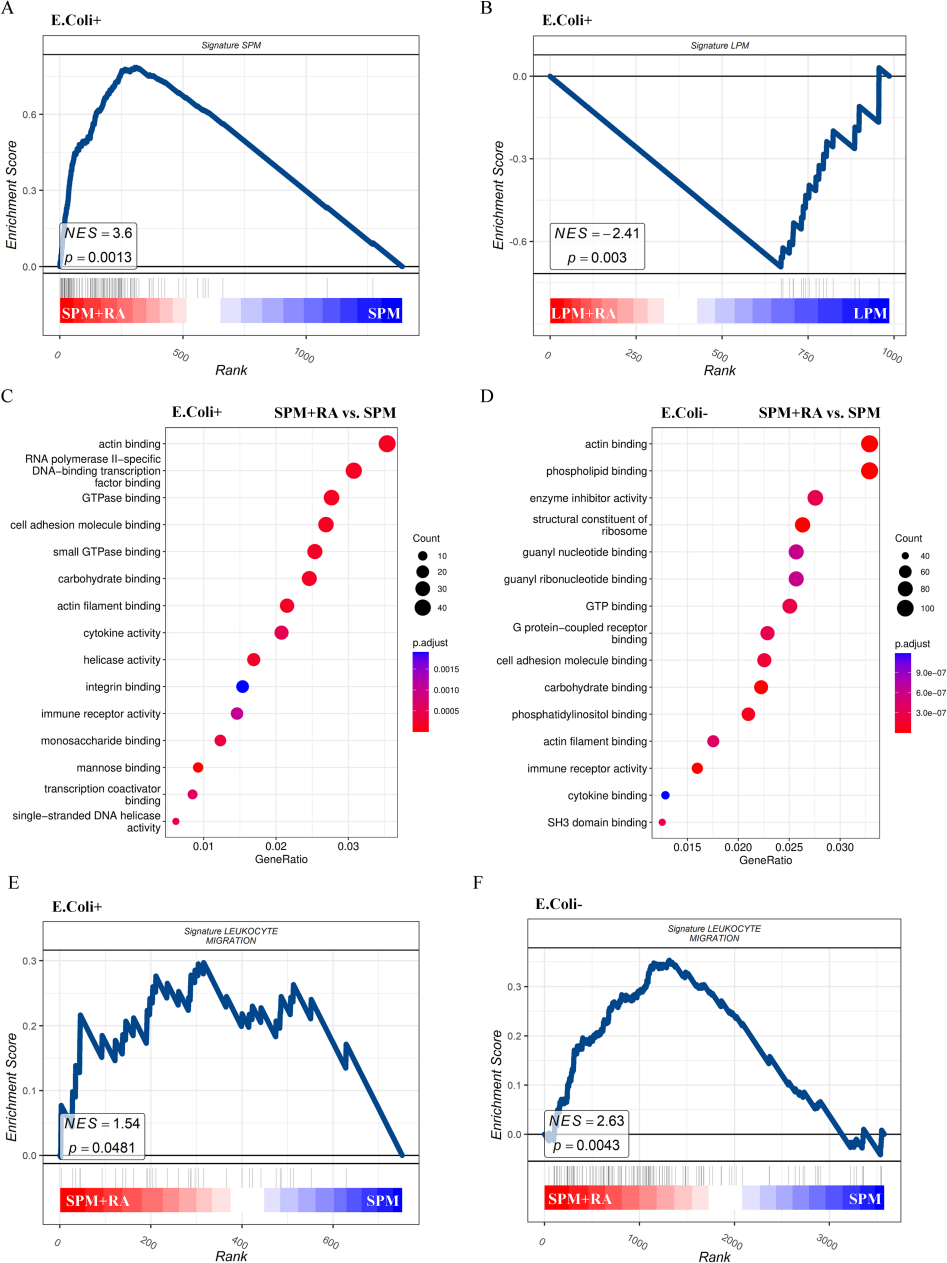
RA treatments promote the expression of genes of SPM signature as well as genes relevant to leukocyte migration. Gene set enrichment analysis (GSEA) visualization of gene signatures of SPM in the *E. coli* phagocytosing SPMs **(A)** and LPMs **(B)** treated with or without RA. GO enrichment analysis in the *E. coli* phagocytosing **(C)** or free **(D)** SPMs treated with vs. without RA. GSEA visualization of gene signatures of leukocyte migration in the *E. coli* phagocytosing **(E)** or free **(F)** SPMs treated with or without RA.

In this study, SPMs and LPMs from the peritoneal cavity of mice were categorized after RA treatment, and RNA sequencing was performed 24 h post-RA treatment to decipher their genome-wide expression. Prior evidence pointed to a varied set of DEGs between SPM and LPMs concerning gene expression, thereby permitting the annotation of selective traits of either SPMs or LPMs genes. The aim of this study was to see if RA influences SPMs and LPMs functional activity via the modification of these engaged gene signatures. Post RNA sequencing analysis denoted that SPMs of the treated group maintained higher expression of “SPMs” gene signatures, while LPMs lost their expression of distinctive “LPMs” genes when treated with RA (Figures 5A,B). This indicates that RA seems to have a regulatory effect on the transcriptome which clearly differs between SPM and LPMs. In order to further characterize RA functional effects on SPMs and LPMs, GO analysis was performed. Contrarily, when compared to untreated LPMs, RA-untreated SPMs significantly express GO functions connected to cell migration and adhesion, such as actin-binding, actin filament binding, small GTPase binding, and integrin binding (Figure 5C). The SPMs-associated cell migration functions were effectively enhanced in the RA treated group; in contrast, no significant upregulation was seen within treated LPMs. This suggests RA might drive SPMs distribution and expansion by encouraging cell migration (Figure 5D). GSEA was therefore performed on metadata of leukocyte-targeted migration genes in RA-treated SPMs blood to test this hypothesis. Analysis of the genes showed that SPMs from the RA-treated group had significant enrichment of leukocyte-targeted migration genes when compared to the untreated SPMs (Figures 5E,F). Probably, such functional differences cannot be linked to the ability of macrophages to phagocytize bacteria and might explain rather the changed distribution of SPMs and their expansion under RA conditions. KEGG results showed (Supplementary Figure 2A) that DEGs were mainly involved in Efferocytosis, cytokine–cytokine receptor interaction, Rap1 signaling pathway, Phagosome, NOD−like receptor signaling pathway. In closing, these data suggest that RA has a profound effect on the differentiation genes and functional state characteristics of SPM and LPM by regulating cell migration and adhesion-related genes. To confirm the involvement of SPM, peritoneal macrophages from mice injected with *E. coli* were analyzed for CD44 and CCR2 flow 24 h after RA pretreatment; CD44 was expressed at a high level up to 30 min before the peak time point, followed by a steady decline. In contrast, there were no significant changes in the control (blank) and RA alone treated groups (Supplementary Figure 2B). CCR2 was not significantly different between the two groups. This suggests that CD44 expression in macrophages increased at 60 min post-infection after 24 h of RA pretreatment, indicating that RA treatment enhanced the time-dependent expression of adhesion molecules on the macrophage surface.

**Figure 6:**
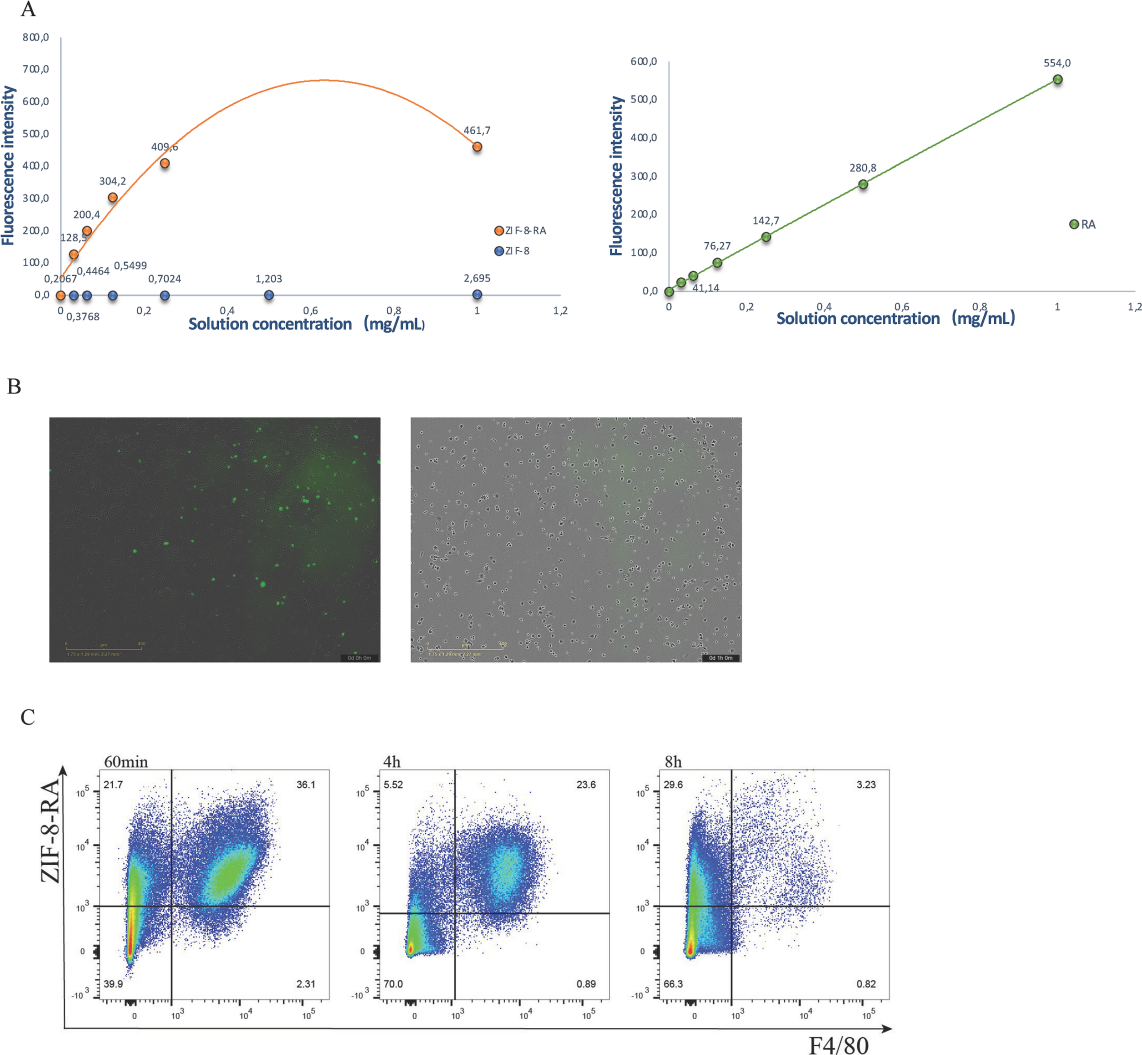
Nanoparticles improved the persistence of RA. **(A)** Fluorescence intensity analysis of ZIF-8 nanoparticles loaded with RA. **(B)** Microscopic imaging of RA-loaded ZIF-8 nanoparticles phagocytosed by peritoneal macrophages (left) and empty ZIF-8 (right) in vitro. **(C)** Time course of peritoneal macrophages that uptake ZIF-8 nanoparticles loaded with RA.

To improve the sustained loading capacity of retinoic acids in macrophages, we designed a drug-loading system based on ZIF-8 nanoparticles (Figure 6A). ZIF-8 is a stabilized metal–organic framework (MOF) that is widely used in delivery systems for its good biocompatibility and drug-loading capacity (Supplementary Figure 2C). In this study, we loaded RA into ZIF-8 with the aim of prolonging its retention time in macrophages and thus overcoming the limitation of rapid metabolism of RA injected alone (Supplementary Figure 2D). We first verified the phagocytosis of ZIF-8 loaded with RA by peritoneal macrophages through *in vitro* experiments (Figure 6B). The results showed that macrophages were able to effectively phagocytose RA-loaded ZIF-8, and the nanoparticles were uniformly distributed in macrophages. Subsequently, we injected RA-loaded ZIF-8 into the abdominal cavity of mice. *In vivo* experiments showed that RA nanoparticles were still stably present in macrophages 4 h after injection, and the proportion of RA-loaded F4/80-positive macrophages was significantly increased compared with that of RA alone. More importantly, a small residue of RA nanoparticles was still detected in F4/80-positive macrophages 8 h after injection (Figure 6C). This result suggests that RA-loaded ZIF-8 nanoparticles significantly prolonged the presence of RA in macrophages compared with the rapid metabolism or clearance of RA injected alone. In conclusion, this study demonstrates the potential of a ZIF-8-based nanoparticle delivery system in prolonging the duration of RA within macrophages. This strategy provides new ideas for the application of RA in inflammation regulation and lays the foundation for future studies on the role of nanoparticle delivery systems in immunotherapy.

## Discussion

Retinoic acid, a known immunomodulator, has been demonstrated to regulate the expression and function of macrophages, playing an integral role in the immune response (6, 7, 17). In this study, we demonstrated the pivotal role of RA in LPMs and *E. coli* infection through the use of immunofluorescence, flow cytometry, and qPCR. It was observed that RA was predominantly distributed in mesothelial and omental tissues and surrounded by F4/80-positive macrophages following the injection of RA (7, 18, 19). This suggests that RA may modulate its antimicrobial capacity by affecting the distribution and function of macrophages in the peritoneal cavity. Furthermore, a 24 h RA injection treatment resulted in a significant prolongation of macrophage phagocytic activity of bacteria, indicating that RA treatment has facilitated bacterial clearance by enhancing the early phagocytic response and rapidly activating macrophage-associated gene expression. In peritoneal macrophages treated with RA, the expression of genes such as Apoc2, Arg1, Cd62p, Lrg1, Rarb, and Thbs1 was highly upregulated. The respective change in expression from these genes would further strengthen the supposed important function of the RA pathway in regard to macrophage metabolism and anti-inflammatory functions (5, 20). In addition, the transcriptome profiles from systematic analysis showed that RA treatment greatly increased the expression of SPMs-identifying genes and was also related to leukocyte migration underlining the importance that RA probably holds in stimulating abdominal immune defense responses through the recruitment of SPMs. With these collective insights, the present major findings uncover how significantly RA controlled phagocytosis by macrophages and altered their numbers and distribution within the peritoneal cavity. Such findings not only broaden the horizon of the understanding of the mechanism of the importance of RA in immunomodulation but also pave the way for new insights and scientific bases for the therapeutic potential of RA in controlling infection.

In the initial stages of bacterial invasion, LPMs rapidly accumulate at the site of infection and eliminate invading pathogens through phagocytosis. However, they are eliminated from the peritoneal fluid when the peritoneum is inflamed (11, 12, 16). Unfortunately, LPMs, while not directly linked to apoptosis, is able to migrate to injured tissues and establish binding interactions. In this aseptic liver injury model, such LPMs migrates to the area of injury, controlling whether the necrotic tissue is bound and thus enhancing the preferred clearance processes. LPMs is one of the mechanisms of defense employed against infection, yet these *E. coli* models or systems in which heparin was injected resulted in increased peritoneal cavity bacterial load in mice (13), with corresponding increases in mortality. Thus, this suggests what could be of potential use in infection management given the fact that there is possible retention or restoration of LPMs function. There is increased resistance to infections with LPMs linked to RA treatment. RA can extend the time for LPMs to remain in peritoneal fluid by modulating their distribution and activity in the early phases of infection and by boosting their phagocytosis and antimicrobial functions. This gives a solid theoretical background for the anticipatory employment of RA in the management and control of inflammatory diseases and infections.

It is proposed that RA may facilitate bacterial clearance by enhancing the early phagocytic response and rapidly activating macrophage-associated gene expression. The modulation of macrophage phagocytosis by RA is consistent with existing literature findings that RA enhances macrophage recognition and clearance of pathogens by regulating the expression of phagocytosis-related genes in the immune response (21). In the RA-treated group of peritoneal macrophages, the upregulation of Arg1 may be associated with the shift of macrophages to an anti-inflammatory phenotype, as evidenced by previous research (22). Furthermore, the up-regulation of Rarb suggests positive feedback regulation of the RA signaling pathway, as observed in other studies (17). In addition, RA has selective effects on small peritoneal macrophages populations. During the early stages of the peritonitis response, when the classical macrophage disappearance response (MDR) leads to LPM depletion, SPMs are recruited to engage in phagocytosis and thus play a key role in maintenance of the peritoneal inflammatory response. Our study further revealed that increased recruitment of SPMs was associated with significant up-regulation of their characterized genes and showed high scores of leukocyte migration related genes by genomic enrichment analysis (GSEA). Among the KEGG pathways it was shown that in Efferocytosis signaling pathway was the most significantly enriched pathway, mainly associated with genes such as Arg1, Mfge8, Alox15, Cx3cr1, etc. Efferocytosis is a process by which macrophages or other macrophage cells remove apoptotic cells by recognizing and removing them, and it is a key mechanism for maintaining tissue homeostasis and immune balance. Apoptotic cells activate the Efferocytosis pathway by releasing inflammatory factors, which can promote M2 macrophage polarization to inhibit the inflammatory response and achieve efficient, anti-inflammatory clearance. Unlike ordinary phagocytosis, it is highly specific and relies on chemokines such as CXCL1 (binding receptor CX3CR1) to attract macrophages to migrate to the vicinity of apoptotic cells to promote phagocytosis initiation (23, 24). This suggests that RA not only plays an important role in enhancing macrophage phagocytosis, but may also modulate peritoneal inflammatory responses by promoting recruitment and functional enhancement of SPMs. In our flow cytometry, we showed an increase in CD44 expression in early macrophages 24 h after RA pretreatment; however, this appeared to be a transient effect. This suggests that RA could act in an immunomodulatory manner to promote the adhesion and migration of macrophages in early infection phases through the CD44 pathway. The majority of CCR2 expression changes were limited, indicating that RA-escorting macrophage modulation was, temporally speaking, mainly dependent on the CD44 pathway. In conclusion, these findings clarify the final mechanism of RA in the modifications of macrophage functioning and outline the temporal features etiological to the pharmacological effects. With corroboration from long-term observational studies, more data from RA would further increase macrophages’ functional status in a more sustained manner.

It was observed that in macrophages treated with RA-loaded ZIF-8 nanoparticles, the nanoparticles were still retained by F4/80-positive cells after 8 h. This is correlated with the feature of ZIF-8 with adjustable sustained release of loaded drugs (25). More interestingly, this prolongation suggests that the nanoparticles not only increased the retention time of RA *in vivo* but may also have altered the dynamic distribution and function of macrophages, which provides new clues for an in-depth study of the role of nanocarriers in immunomodulation.

In conclusion, our data provide a more in-depth theoretical basis for understanding the regulatory role of RA in infection and inflammation, as well as new ideas for developing therapeutic strategies targeting RA. However, it should be noted that this study has certain limitations, On the whole such limitations do not interfere with the scientific value of this study; instead, they should provide a precise path for the improvement of future studies and move the application of RA forward within infection control and immunotherapy. RA has very relevant effects in the regulation of peritoneal macrophage function, and the RA delivery system demonstrated is a promising candidate for prolonging the bioavailability of the active drug. To cite some possible limitations: The investigations performed used a single animal model of peritonitis and an *in vitro* macrophage culture system which somehow do not allow a full representation of the varied pathological mechanisms behind human peritonitis. This study has been quite limited in the evaluation of the respective effect of RA over the long run in regulating innate immune responses during various stages of inflammation, as well as the control spanning over limited time intervals (60 min, 4 h, and 24 h). Besides, the specific distinction of LPMs from SPMs has not yet been finely analyzed nor have we independently assessed the expression of genes representing LPMs and SPMs in qPCR: this will affect our understanding of specific RA regulatory roles across different macro-phage subpopulations. In our ZIF-8 nano-delivery system, retention studies on a longer duration for RA in macrophages have not been performed, and still, we are under an open question about the stability of nanoparticles in complex vivo environments. Future studies are expected to fully optimize the nanoparticle-loaded drug regimen and to evaluate the scope of that treatment in other infection, or inflammation models. After taking into account those limitations, we aim to perform dynamic sequencing on multiple models/time points for single-cell monitoring. We shall further see the in-depth system to realize RA-modulated different macrophage subpopulation modes during peritonitis and allergenic strife. Also, this would further bolster robust nanocarrier delivery systems, and enhance the stability and clinical prospect of RA.

## Data Availability

The datasets presented in this study can be found in online repositories. The names of the repository/repositories and accession number(s) can be found in the article/Supplementary material.

## Supplementary material

SUPPLEMENTARY FIGURE 1 Immunofluorescence analysis of phagocytosis of RA and *E. coli* and macrophage aggregation on peritoneal mesothelium and greater omentum at different scales. **(A)** Immunofluorescence images of peritoneal mesothelial tissue showing the distribution of F4/80 staining (white), RA (green) and *E. coli* Tdtomato (red). **(B)** Immunofluorescence images of the greater omentum with F4/80 staining, RA and *E. coli* Tdtomato.

SUPPLEMENTARY FIGURE 2 **(A)** KEGG pathway enrichment analysis of LPM and SPM after RA treatment. **(B)** FACS analysis of CCR2 and CD44 expression in mice after intraperitoneal RA treatment. **(C)** Morphology of ZIF-8 under transmission electron microscope. **(D)** High performance liquid chromatography showed that ZIF-8 was loaded with retinoic acid, and ZIF-8 particles successfully encapsulated retinoic acid in 8 min, and the separation effect was well.

SUPPLEMENTARY FIGURE 3 Integrated technical workflow for RA-related macrophage study.
