# Correction: Retinoic acid modulates peritoneal macrophage function and distribution to enhance antibacterial defense during inflammation

**DOI:** 10.3389/fnut.2025.1746132

**Published:** 2025-12-04

**Authors:** Yujuan Qin, Xi Wang, Xiamin Zhang, Lianting Nong, Qiyan Hou, Yuhong Chen, Yuting Li, Wenxian Lin, Xiuli Mao, Kezhao Wu, Wenqian Nong, Tonghua Wang, Lingzhang Meng, Jian Song

**Affiliations:** 1 Graduate School, Youjiang Medical University for Nationalities, Baise, China; 2 Institute of Cardiovascular Sciences, The People's Hospital of Guangxi Zhuang Autonomous Region & Guangxi Academy of Medical Sciences, Nanning, China; 3 School of Medicine, Guangxi University, Nanning, China; 4 Department of Digestive Diseases, Affiliated Hospital of Youjiang Medical University for Nationalities, Baise, China; 5 Institute of Physiological Chemistry and Pathobiochemistry, University of Münster, Münster, Germany

**Keywords:** peritonitis, large peritoneal macrophages, small peritoneal macrophages, retinoic acid, nanoparticles

An incorrect number was provided for the Natural Science Foundation of Guangxi Zhuang Autonomous Region. The correct number is 2023GXNSFAA026041.

The original version of this article has been updated.

