# Supplementary figures and images for: Retinoic acid modulates peritoneal macrophage function and distribution to enhance antibacterial defense during inflammation
Source: Front Nutr. 2025 Apr 30;12:1545720. doi: 10.3389/fnut.2025.1545720 (PMC12075188; doi:10.3389/fnut.2025.1545720)

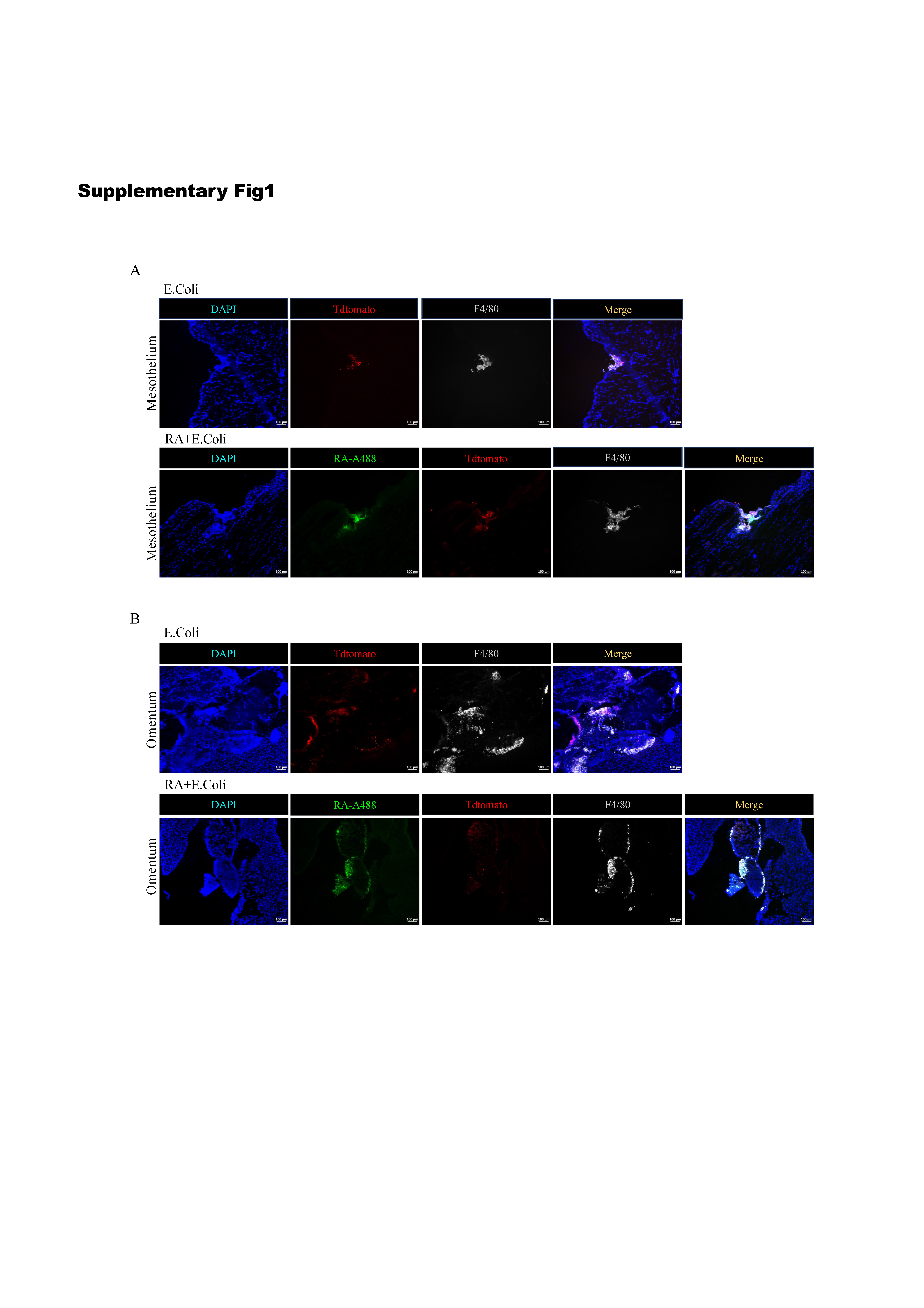

Supplement: SUPPLEMENTARY FIGURE 1 — Immunofluorescence analysis of phagocytosis of RA and E. coli and macrophage aggregation on peritoneal mesothelium and greater omentum at different scales. (A) Immunofluorescence images of peritoneal mesothelial tissue showing the distribution of F4/80 staining (white), RA (green) and E. coli Tdtomato (red). (B) Immunofluorescence images of the greater omentum with F4/80 staining, RA and E. coli Tdtomato. [file Image_1.tiff]

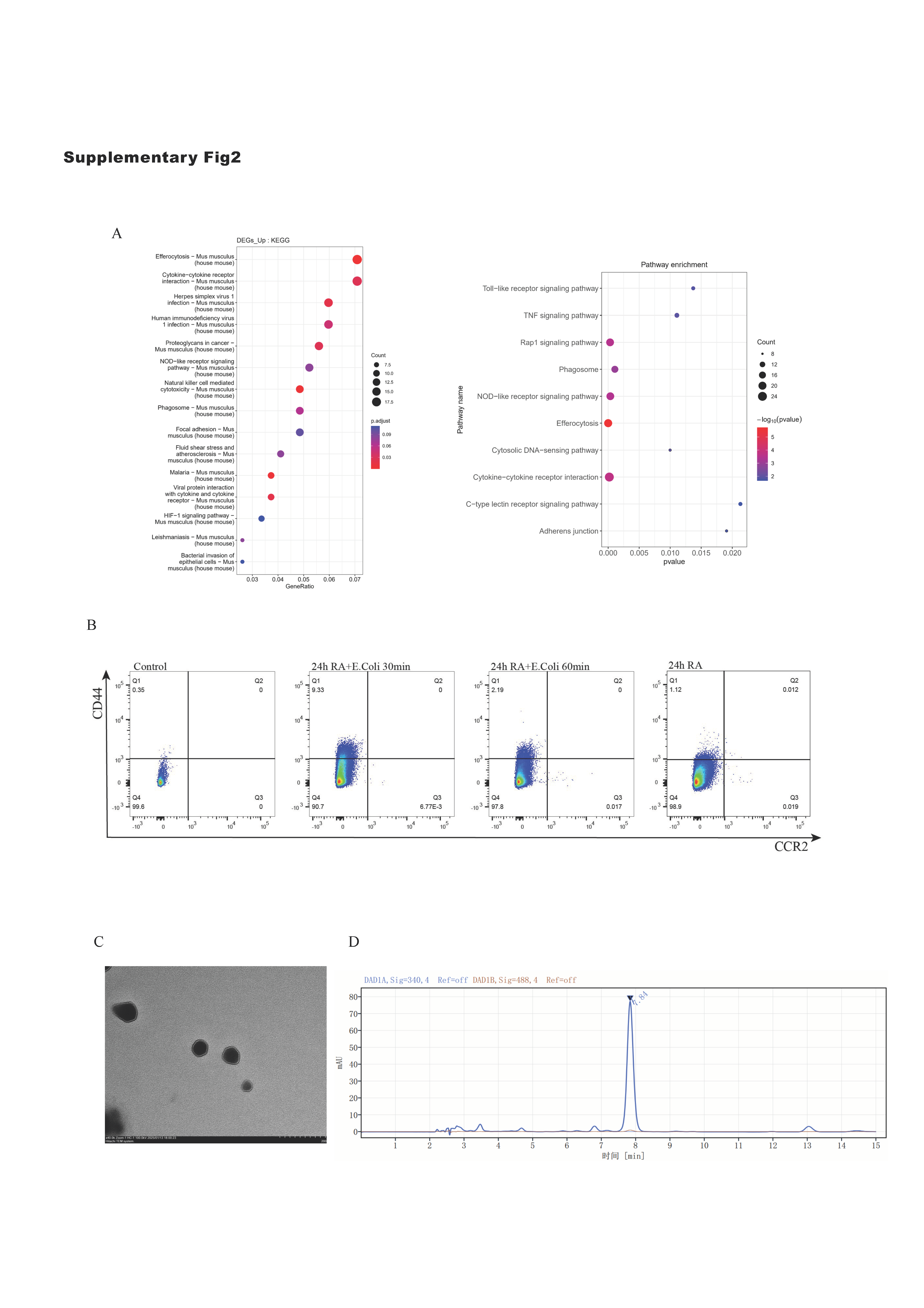

Supplement: SUPPLEMENTARY FIGURE 2 — (A) KEGG pathway enrichment analysis of LPM and SPM after RA treatment. (B) FACS analysis of CCR2 and CD44 expression in mice after intraperitoneal RA treatment. (C) Morphology of ZIF-8 under transmission electron microscope. (D) High performance liquid chromatography showed that ZIF-8 was loaded with retinoic acid, and ZIF-8 particles successfully encapsulated retinoic acid in 8 min, and the separation effect was well. [file Image_2.tiff]

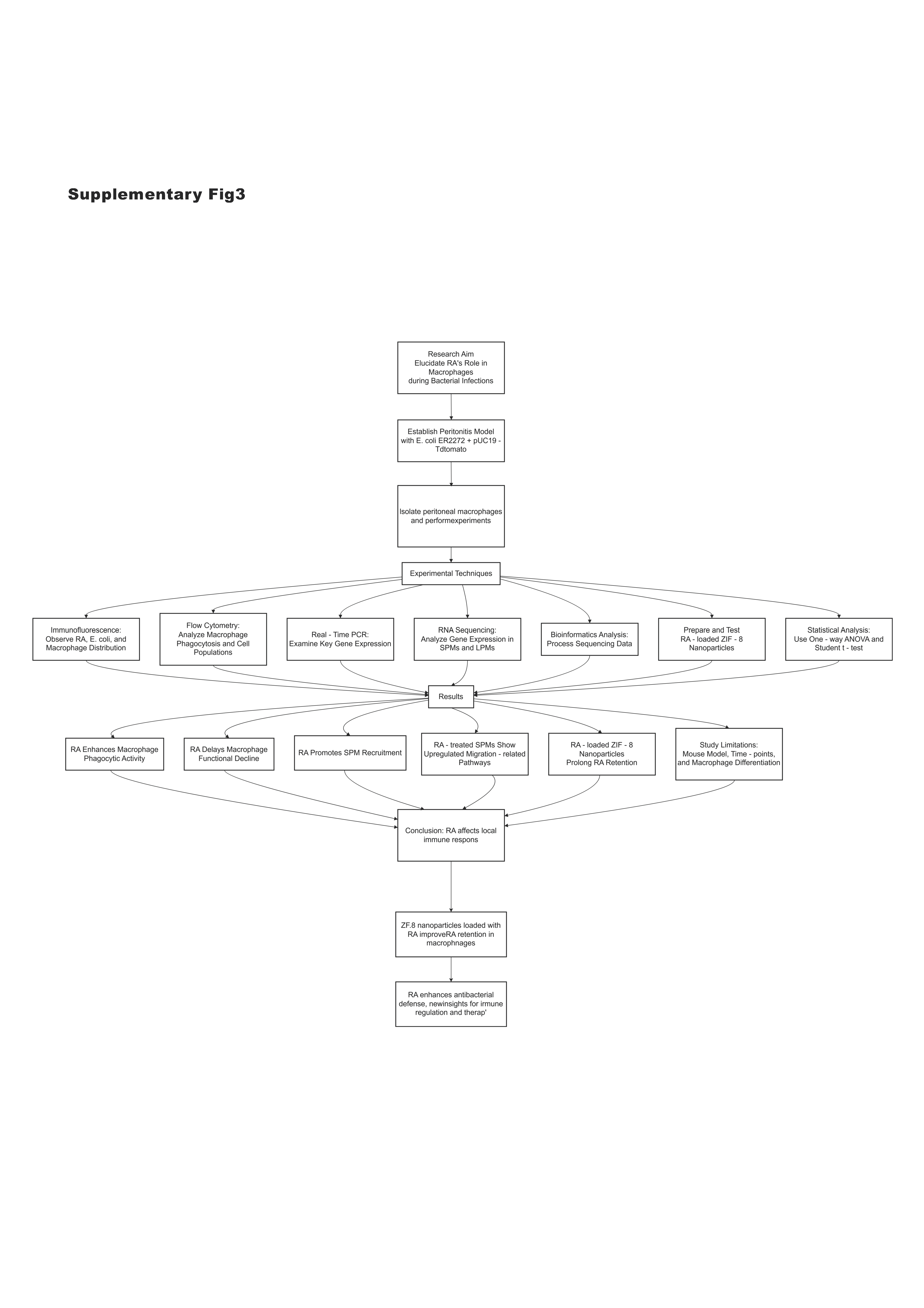

Supplement: SUPPLEMENTARY FIGURE 3 — Integrated technical workflow for RA-related macrophage study. [file Image_3.tiff]
